# Knowledge on Antibiotic Use, Self-Reported Adherence to Antibiotic Intake, and Knowledge on Multi-Drug Resistant Pathogens – Results of a Population-Based Survey in Lower Saxony, Germany

**DOI:** 10.3389/fmicb.2019.00776

**Published:** 2019-04-12

**Authors:** Heike Raupach-Rosin, Nicole Rübsamen, Gesa Schütte, Gabriele Raschpichler, Pa Saidou Chaw, Rafael Mikolajczyk

**Affiliations:** ^1^Research Group Epidemiological and Statistical Methods, Department of Epidemiology, Helmholtz Centre for Infection Research, Braunschweig, Germany; ^2^PhD Programme “Epidemiology”, Braunschweig–Hannover, Germany; ^3^Institute of Medical Epidemiology, Biometry and Informatics, Medical Faculty, Martin-Luther-University Halle-Wittenberg, Halle, Germany

**Keywords:** adherence, antibiotic use, antibiotic resistance, compliance, MDR-pathogens, public knowledge

## Abstract

**Introduction:**

Assessment of public awareness on antibiotic use and resistance can identify key issues for campaigns addressing these problems. Our aim was to assess the knowledge, attitudes, and practice (KAP) related to antibiotic use and multi-drug resistant (MDR) pathogens in a general population in Germany.

**Methods:**

We conducted a KAP survey on antibiotics and on MDR pathogens using an online panel recruited from the general population, which was established using stratified random sampling from the population registry in four districts in Lower Saxony, Germany.

**Results:**

In the 12 months preceding the survey, 32.3% of the participants had received at least one prescription for antibiotics, 95.7% reported to follow the recommendations of prescribers, and 10.3% reported to stop taking antibiotics as soon as they feel better. Up to 94.9% of the participants had heard of MDR pathogens, 42.7% reported to know somebody who had been tested positive for it, 0.8% had an infection with it, and 37.2% were worried of contracting it. In case of contact with a carrier of MDR pathogens, over 90% would increase hand hygiene and 0.8% would avoid the carrier completely. Participants considered health care workers (75.1%) and everybody in society (87.8%) to be responsible for combating the spread of MDR pathogens.

**Conclusion:**

There is a high reported exposure to antibiotics and awareness of the problem of MDR pathogens. Despite personal worries, most of the participants indicated a reasonable, non-stigmatizing behavior toward carriers of MDR pathogens, and that every individual was responsible to avoid their spread.

## Introduction

The development of antibiotic resistance is a major public health concern in Europe (European Centre for Disease Prevention and Control, 2017) and worldwide (World Health Organization [WHO], 2014). Antibiotic resistances cause longer hospital stays, increased mortality (Ott et al., 2010), and substantial economical and intangible costs (Ott et al., 2010; Claus et al., 2014; Raupach-Rosin et al., 2015, 2016). Understanding the current public knowledge about antibiotics and the development of resistances and identifying misconceptions could help to shape policies and campaigns addressing these problems (Shallcross et al., 2015; World Health Organization [WHO], 2015).

The World Health Organization [WHO], 2015 multi-country report showed that awareness of the existence of antibiotic resistance was widespread in general populations, but understanding of the implications was mixed (World Health Organization [WHO], 2015). Adherence to antibiotic treatment is not only important to ensure the therapeutic effect, but also to prevent the development of antibiotic resistances. Studies from the United Kingdom and Italy showed that early termination of antibiotics was a common problem, and happened mostly when patients started to feel better (McNulty et al., 2007; Grosso et al., 2012).

Despite available data on public perceptions regarding antibiotic use in the EU member states (McNulty et al., 2007; Grosso et al., 2012; McCullough et al., 2016; Mazińska et al., 2017), generalization of these results may be difficult since antibiotic use is influenced by several factors such as mode of availability and accessibility, national policies, amongst other factors (Zajmi et al., 2017). Furthermore, some of the studies included participants who were either recruited from health facilities (Grosso et al., 2012), or have had some form of interventional awareness program (Mazińska et al., 2017), thus may not been representative of the general population. Although a study from the United Kingdom included the general population (McNulty et al., 2007), information on the effect of factors such as the source of information and level of exposure to antibiotics, on the perceptions of antibiotic use was lacking. These factors could influence the mode and outcome of possible interventional programs that can be useful in promoting public awareness on antibiotic use. On the other hand, there is limited data on public perceptions about antibiotic resistance in the EU; those available (Easton et al., 2009; McCullough et al., 2016; Vallin et al., 2016), focused more on the general understanding of antibiotic resistance but to a lesser extent on its implications.

Therefore, our aim was to assess the current public knowledge about antibiotic use and its implications for antibiotic resistance development in Germany. We also aimed to find out whether differences exist in the general perceptions of the different sub-groups of the population (age group, sex, work, level of education, and sources of self-information about antibiotic resistance) and the impact of these factors on public knowledge about antibiotic use and multi-drug resistant (MDR) pathogens.

## Materials and Methods

### Participants

We conducted a KAP survey on antibiotic use and MDR pathogens using a longitudinal online panel that was initiated in March 2014 to address hygiene and preventive behavior regarding infectious diseases (HaBIDS) as described elsewhere (Rübsamen et al., 2017a,b). The panel was established using stratified random sampling from the population registry in four districts in Lower Saxony, Germany (Braunschweig, Salzgitter, Vechta, and Wolfenbüttel).

### Questionnaires

The panel members received the questionnaire addressing their KAP on antibiotic use in February 2015. The KAP on antibiotic use covered three topics: exposure to antibiotics, assessed by asking if and for which symptoms panel members had been prescribed antibiotics for the preceding 12 months (two items); knowledge about antibiotics, assessed using statements relating to correct identification of antibiotics and opinions regarding antibiotic use in general (seven items: questions 1–7); and attitudes and practice toward antibiotic use, assessed using statements regarding how participants generally obtain antibiotics for themselves or a family member when they are ill, and if they are concerned about antibiotic resistance (seven items: questions 8–14), and questions and statements regarding the last time a panel member had received antibiotics, whether they asked for them, how they used them, and whether they developed any side effects during use (six items: questions 15–20) [see Supplementary Material – German questionnaires (used to conduct the surveys)].

In November 2015, panel members received a second KAP questionnaire addressing MDR pathogens. The questions covered four topics: personal exposure to MDR which was assessed by asking if and where panel members had heard about MDR and if they themselves or someone they knew had been diagnosed with MDR in the past (seven items: questions 1–6, and 15); knowledge on MDR pathogens, assessed by using questions and statements regarding how one can get infected with an MDR pathogen, its spread, and the treatment options (five items: questions 10–14); attitudes and practice regarding MDR pathogens, assessed by asking participants about worries for themselves or a family member with respect to contracting a MDR pathogen, about MDR pathogens being a problem of society as a whole, and their opinion on who is responsible for the control of their spread (ten items: questions 7–9, and 16–22), and by using typical scenarios of how participants will react toward a close contact having an infection with an MDR pathogen (two items: questions 23–24). Information on sociodemographic characteristics was collected through a separate questionnaire in HaBIDS and included in our analysis. For the development of questionnaires related to antibiotic use or MDR pathogens, we followed ideas and questions from already published qualitative and quantitative studies (Mattner et al., 2006; Brinsley-Rainisch et al., 2007; Easton et al., 2009; Ling Oh et al., 2011; Wun et al., 2013). The translated original questionnaires are available as online supplement [see Supplementary Material – English translations of the German questionnaires (not validated for use in the surveys)].

### Data Analysis

**FIGURE 1:**
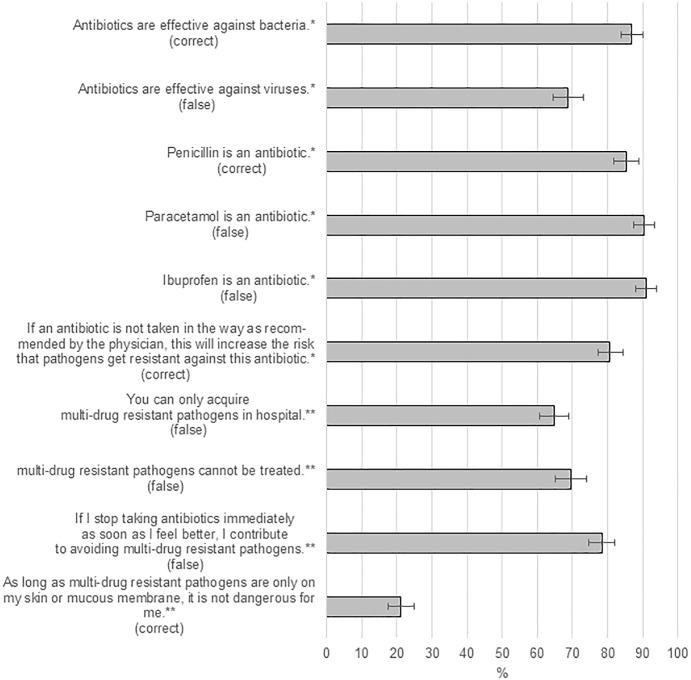
Knowledge of the participants: Percentages of correct answers to each question (assessed using post-stratification to obtain estimates representative for the general population of Lower Saxony). ^∗^Items from the survey on antibiotics. ^∗∗^Items from the MDR survey. “Correct” means that the statement in itself is correct and should be agreed with by the participants to count as correct answer. “False” means the statement in itself is wrong and should be disagreed with by the participants to count as correct answer.

We restricted the data analysis to panel members who filled in both questionnaires. To grade the level of knowledge, we computed a cumulative score on six of the knowledge items from the survey on antibiotics and four of the items from the MDR survey (1 point for each answer in agreement with current scientific knowledge, range 0–10), Figure 1. We did not include the seventh knowledge item from the antibiotics survey (“Individuals who take antibiotics regularly have a higher risk that their body becomes immune against antibiotics”) in the score because several participants stated in the comments that this question was misleading.

Data was analyzed descriptively using post-stratification (Lumley, 2010) to obtain estimates representative for the general population of Lower Saxony. We used age, sex, and highest completed educational level to generate the weights for post-stratification. Population level data were taken from the German census 2011 (Federal Statistical Office of Germany, 2011). To make comparisons between groups of participants, we used χ^2^ tests for the categorical variables (age group, sex, employment in a medical sector or not, level of education, and sources of self-information about antibiotic resistance). We performed explorative linear multivariable regression analyses (ANCOVA) to assess the effect of these factors on knowledge about antibiotics and MDR pathogens using the knowledge score as outcome variable. The model was built using manual backward selection (Hosmer and Lemeshow, 2005) and complete case analysis. No weighting was used in regression analyses. Variables with *p* < 0.25 in the univariable analysis were included in the model building procedure (using as cut-off *p* ≥ 0.05 for exclusion, based on the likelihood-ratio test).

We tested the variables included in multivariable models for multicollinearity using the variance inflation factor (O’brien, 2007). In addition to the regression coefficients and the 95% confidence intervals (CI), the effect size was calculated as a measure of how much variation in knowledge could be explained by the given variable. The effect sizes were expressed as partial η^2^. A partial η^2^ ≥ 0.01 and <0.06 presents a small effect, ≥0.06 and <0.14 presents a medium effect, ≥0.14 presents a strong effect (Kotrlik et al., 2011). Analyses were performed in Stata version 12 (StataCorp, 2011) and R version 3.5.1 (R Core Team, 2018). We considered *p* < 0.05 as significant.

### Ethics Statement

This study was approved by the Ethics Committee of the Hannover Medical School (No. 2021-2013) and the Federal Commissioner for Data Protection and Freedom of Information. All participants gave written informed consent before entering the study.

## Results

### General Characteristics of the Respondents

Out of the 1,037 panel members, 977 (94.2%) completed the questionnaire on antibiotic use, 912 (87.9%) on MDR pathogens, and 872 (84.1%) filled in both questionnaires. The participants who filled both questionnaires were older than those who missed at least one of the two questionnaires (median 50 vs. 45 years, *p* < 0.001). There was no significant difference between the two groups in terms of sex or education. More than half (60.2%) of the participants were female and nearly 10% worked in the medical sector (Table 1).

**Table 1:** Characteristics of the participants.

	Participants (*N* = 872)
**Age,** median (1st quartile, 3rd quartile)	50.0 (38.0, 59.0)
**Sex,** N (%)	
Female	525 (60.2%)
*Sex is missing^a^*	0
**Highest completed educational level,** N (%)	
Lower secondary education or apprenticeship	239 (28.1%)
Still at upper secondary school	19 (2.2%)
University entrance qualification (upper secondary education or vocational school)	229 (26.9%)
University degree	364 (42.8%)
*Highest completed educational level is missing^a^*	21
**Marital status,** N (%)	
Unmarried	244 (28.7%)
Married	517 (60.9%)
Divorced or widowed	88 (10.4%)
*Marital status is missing^a^*	23
**Employment in the medical sector,** N (%)	
Yes	63 (9.9%)
No	574 (90.1%)
*Employment in the medical sector is missing^a,b^*	235
**Self-rated health,** N (%)	
Poor	4 (0.5%)
Fair	56 (6.6%)
Good	412 (48.9%)
Very good	307 (36.4%)
Excellent	64 (7.6%)
*Self-rated health is missing^a^*	29


*^*a*^Relative frequency was calculated without missing values. ^*b*^The question about employment in the medical sector was part of another online questionnaire in the panel. Because of the survey design, 224 participants were not invited to fill in this particular online questionnaire so that the information about employment in the medical sector is missing. (These 224 participants used to fill in paper-and-pencil questionnaires at the time that this particular online questionnaire was sent).*

### Exposure to Antibiotics and MDR Pathogens

One third of the participants (32.3% [95% confidence interval 27.7%, 37.0%]) had received at least one prescription for antibiotics in the 12 months preceding the first survey, with a significantly higher proportion of the participants under 40 years (39.2% [30.4%, 48.1%]) compared to those older (28.1% [23.1%, 33.2%]), *p* < 0.001. Half of those with prescription (48.2% [39.7%, 56.7%]) reported symptoms of respiratory tract infection as indication for the prescription of antibiotics. Almost all participants (94.9% [92.2%, 97.5%]) had heard of MDR pathogens, 42.7% [38.0%, 47.3%] reported to know somebody who was tested positive for an MDR pathogen, and 0.8% [0.2%, 1.4%] reported that they were at some point in their life tested positive for an MDR pathogen.

### Knowledge About Antibiotics and MDR Pathogens

Most of the respondents correctly answered the questions regarding what antibiotics are, about half of the respondents correctly answered the questions concerning antibiotic resistance and its consequences, Figure 1.

In the multivariable analysis, predictors for good knowledge (measured as knowledge score including both the knowledge about antibiotics and MDR pathogens) were having university education, employment in the medical sector, and self-information through newspapers or internet (Table 2).

**Table 2:** Predictors of knowledge about antibiotics and multi-drug resistant pathogens, Lower Saxony, Germany, 2015.

	Regression coefficient^a^ (95% CI)	*P*-value	Partial η^2^
**Highest completed educational level**			
Lower secondary education or apprenticeship	-0.96 (-1.27; -0.66)	<0.001	5.1%
Still at upper secondary school	-0.66 (-1.76; 0.44)	0.240	
University entrance qualification (upper secondary education or vocational school)	-0.33 (-0.62; 0.03)	0.031	
University degree	Reference^b^		
**Are you employed in the medical sector and do you have contact with patients?**			
Yes	1.23 (0.81; 1.65)	<0.001	4.5%
No	Reference^b^		
**Where have you heard something about MDR pathogens?**			
**Newspaper**			
Yes	0.37 (0.04; 0.70)	0.03	1.2%
No	Reference^b^		
**Internet**			
Yes	0.45 (0.19; 0.70)	0.001	2.1%
No	Reference^b^		


*^*a*^Multivariable linear regression (complete case analysis including data of 602 participants). ^*b*^Reference, this is the reference category of the linear regression model.*

### Attitudes and Practice Toward Antibiotic Use and MDR Pathogens

Some (6.3% [4.1%, 8.5%]) of the participants reported that they had already asked their general practitioner for a prescription of antibiotics while having symptoms of a common cold at some point. Regarding adherence to the recommended regimen of antibiotics, the vast majority (95.7% [93.5%, 98.0%]) reported to take antibiotic as recommended by the physician or pharmacist. In contrast, 10.3% [7.0%, 13.5%] reported to stop taking antibiotics as soon as they feel better (Figure 1). Male participants answered this more frequently than females (15.1% [9.5%, 20.7%] vs. 5.6% [2.2%, 8.9%], *p* < 0.001) as well as participants under 40 years compared to older participants (15.0% [8.0%, 22.0%] vs. 7.4% [4.3%, 10.4%], *p* < 0.001). Participants rarely shared medications with their family members (0.4% [0.0%, 0.8%]) or had antibiotics at home to use when necessary (2.0% [0.9%, 3.2%]).

The topic MDR pathogens was perceived as important, 61.1% [56.5%, 65.6%] considered it to be very important. Participants over 40 years reported significantly more frequently than those under 40 years to be very or rather worried about the risk of getting MDR pathogens themselves (41.9% [36.5%, 47.3%] vs. 29.5% [22.8%, 36.1%], *p* = 0.006) or others in the society (52.1% [46.6%, 57.5%] vs. 38.6% [31.1%, 46.2%], *p* = 0.006), but not about the risk of their family members getting MDR pathogens (47.6% [42.1%, 53.0%] vs. 42.6% [33.7%, 51.5%], *p* = 0.36). Many (78.5% [74.7%, 82.2%]) of the participants correctly disagreed with the statement that “if I stop taking antibiotics immediately as soon as I feel better, I contribute to avoiding multi-drug resistant pathogens.” In order to assess how participants would deal with carriers of MDR pathogens in daily life, we used two scenarios. The first described an old neighbor having an infection with an MDR pathogen in hospital, and the second one described a colleague being tested positive for an MDR pathogen. In both scenarios, the majority (96.7% [94.7%, 98.8%] and 90.0% [86.8%, 93.2%]) would employ good hand hygiene after contact, only few (0.8% [0.2%, 1.4%]) would avoid the neighbor completely or ask their superior to be transferred to another room (5.7% [3.7%, 7.7%]). About one third indicated that they would not allow their children to visit the neighbor anymore (29.9% [25.5%, 34.3%]) with no significant difference between the groups. About one third would be afraid of attracting the pathogens (38.1% [33.3%, 42.8%]).

Regarding limiting the spread of MDR pathogens in the health care sector, 75.1% [70.7%, 79.4%] of the participants considered health care workers, 87.8% [84.8%, 90.9%] every individual, and 58.3% [53.5%, 63.2%] politicians as being responsible for this. Responsibility for reduction of antibiotic use in livestock breeding was attributed to farmers (68.2% [63.6%, 72.9%]), politicians (42.1% [37.2%, 47.0%]), and consumers (53.9% [49.0%, 58.7%]). Many participants (73.0% [68.6%, 77.4%]) agreed that they would be willing to spend more money on meat, if this could reduce antibiotic use in livestock breeding.

## Discussion

We investigated perceptions related to antibiotic use and MDR pathogens in the public in Germany by surveying participants of the online panel HaBIDS. Participants showed a good knowledge about antibiotics and good adherence to its use. Awareness about MDR pathogens was high, but knowledge about antibiotic resistance, MDR pathogens, and their consequences was more limited.

About one third of the participants had received antibiotics within the year before the survey, with younger people being treated more often. Thus, use of antibiotics was common in the study population. Similarly, McNulty et al. reported that 38% of adult participants in the United Kingdom took antibiotics during the last year (McNulty et al., 2007), and in a report of a German health insurance company from 2014, 28% of the male and 40% of the female participants reported intake of antibiotics within the last year (DAK Forschung, 2014). The main indications for antibiotic prescription in our study were upper respiratory tract infections, which is similar to results of the German health insurance company report (DAK Forschung, 2014). As the majority of those infections are caused by viral agents, international recommendations suggest a restrictive use of antibiotics in order to diminish resistance development and prevent adverse effects as well as unnecessary cost (Centre for Clinical Practice at NICE, 2008; Zoorob et al., 2012). Thus, there still seems to be a considerable need for reducing antibiotic use in Germany.

Although 21.5% of the participants falsely agreed with the statement that “if I stop taking antibiotics immediately as soon as I feel better, I contribute to avoiding multi-drug resistant pathogens,” only about ten percent actually indicated to stop taking antibiotics as soon as they feel better. However, a previous study reported a higher rate of premature discontinuation of antibiotic therapy for the general adult population in United Kingdom (11.3%) compared to our study (McNulty et al., 2007). In a study of pediatric patients in Germany, adherence to the antibiotic regimen was measured by analyzing the concentration of the antibiotics in the children’s urine on the last day of planned therapy which was positive in only 60.5% (Hoppe et al., 1999). Thus, our results indicate that premature discontinuation of antibiotic therapy in German adults might be less common than in the general population in the United Kingdom, and in German pediatric patients, but it is possible that the participants in our study overestimated their adherence. Interestingly, an increasing number of studies have shown that treatment durations can be shortened in comparison to initial recommendations, e.g., for community acquired pneumonia (Pinzone et al., 2014; Uranga et al., 2016). Data supporting shorter treatment durations is growing, but there is still need for further research on the optimal duration of antibiotic treatment. Thus, shortening the use of antibiotics could be a useful method to improve patient adherence.

Almost all participants had heard about MDR pathogens. Similarly, Easton et al. (Easton et al., 2009) reported in their Scottish study that 86% of participants had heard of Methicillin-resistant *Staphylococcus aureus* (MRSA). In our study, a surprisingly high number (42.7% of participants) reported to know somebody diagnosed with an infection with MDR pathogens, which could have influenced the level of knowledge. On the other hand, Easton et al. (2009) also reported that 32% of their participants knew somebody with MRSA, but they interviewed hospital visitors. These numbers show that the topic of MDR pathogens personally affects a large part of the general public.

Education level, employment in the medical sector, and self-information from internet or newspapers had positive significant effect on knowledge about antibiotics and MDR pathogens in our multivariable analysis. Education level and employment in the medical sector were shown to have the strongest effect (partial η^2^ of 5 and 4.5%, respectively). This should be kept in mind when making information for the general public available, with regard to both comprehensibility and the choice of the medium. Gaps in specific knowledge about the implications of MDR pathogens, e.g., existing treatment possibilities, were shown before (Mattner et al., 2006; Easton et al., 2009), thus, knowing the right target population and using the right media will be useful when giving information that can help improve the knowledge of the public on the proper use of antibiotics so as to limit the consequences of its misuse such as the spread of MDR pathogens.

One third of the respondents reported being worried personally about getting infected with an MDR pathogen; nonetheless, the answers to the scenarios showed that the majority of participants indicated a reasonably, non-stigmatizing behavior against carriers of MDR pathogens in their neighborhood. This is a remarkable finding, considering the fact that more than one third of the participants stated to be afraid of getting MDR pathogens themselves. However, a small number of participants would adopt a more stigmatizing behavior like changing room or not visiting the neighbor anymore. This result is in line with findings from a survey among MRSA carriers where only 8.5% reported to feel avoided in private settings, whereas they more frequently showed a self-stigmatizing behavior (17.4%) (Raupach-Rosin et al., 2016).

As in other studies, most participants considered health care workers and politicians responsible for dealing with the problem of MDR pathogens (McCullough et al., 2016). But in our study, almost 90% of the participants considered “every individual” responsible as well.

### Strengths and Limitations

The strength of our study is the comparably large sample size and the fact that the study population is sampled from the general population and not from hospital visitors or students like in former studies on this subject.

Our study also has some limitations as follows: The generalisability of the findings may be affected by the higher percentage of individuals holding a university degree compared to the source population (42.8% in HaBIDS vs. 13.5% in Lower Saxony).

Not all the panel population filled in both questionnaires (antibiotic use and MDR pathogens). Although a high proportion filled in both (84.1%), it is reasonable to assume a bias toward people with a higher interest in this specific topic, probably those with higher knowledge and/or personal experience with antibiotics or resistant bacteria. However, there was no difference in the sociodemographic characteristics of the two groups.

Information on medical profession was missing for 25.6% of the participants, but since this information is missing completely at random (due to organizational reasons, not all participants where asked the question about medical profession), the complete case approach for linear regression is still valid.

Because the knowledge on antibiotics and MDR pathogens was assessed by closed questions, respondents may have chosen the most favorable answers, thus, a qualitative approach might be more suitable to reveal misconceptions.

## Conclusion

General knowledge about antibiotics was good but knowledge regarding antibiotic resistance, MDR pathogens, and their implications was less pronounced. However, there was a high awareness about antibiotic resistance being a problem and a considerable proportion reported to be personally affected by multidrug resistances. Therefore, information about the implications of MDR pathogens should be made available for the general public. Policy campaigns about the correct use of antibiotics could increase knowledge and thereby improve appropriate practice in regard to the problem of antibiotic use and resistance in both humans and livestock settings in the German public. Thus, in addition to the already provided new information from our results which could be useful to policy makers, the data could serve as a basis for development of interventional programs.

## Ethics Statement

This study was approved by the Ethics Committee of the Hannover Medical School (No. 2021-2013) and the Federal Commissioner for Data Protection and Freedom of Information. All participants gave written informed consent before entering the study.

## Author Contributions

HR-R and NR conceived the study. HR-R developed the questionnaires. NR conducted the survey. NR and GS performed the statistical analysis. PC contributed on the statistical analysis and interpretation of the results. RM provided technical expertise and advice on conducting the survey and on the interpretation of the data. All authors contributed to the manuscript and equally responsible for the content of the manuscript and have read and approved the final manuscript.

## Conflict of Interest Statement

The authors declare that the research was conducted in the absence of any commercial or financial relationships that could be construed as a potential conflict of interest.
