# Supplementary material for: Knowledge on Antibiotic Use, Self-Reported Adherence to Antibiotic Intake, and Knowledge on Multi-Drug Resistant Pathogens – Results of a Population-Based Survey in Lower Saxony, Germany
Source: Front Microbiol. 2019 Apr 12;10:776. doi: 10.3389/fmicb.2019.00776 (PMC6473076; doi:10.3389/fmicb.2019.00776)
Supplement: Supplementary file 1 [file Data_Sheet_1.PDF]

III. ANTIBIOTICS

In this part of the questionnaire we would like to enquire from you about the intake of antibiotics. Antibiotics are medicaments which brake or kill infectious agents in their growth. Infections are caused by pathogens and are often accompanied by fiver.

In Germany antibiotics are available only on prescription and have to prescribe by a doctor.

Please remember in the last twelve month, did you get an antibiotic prescription?

☐ No

☐ Once

☐ Two to three times

☐ More than four times

☐ Do not know

Which symptoms did you have at the time? (if you had several antibiotic prescriptions, please think about the last time) (Multiple responses are possible)

☐ Cough

☐ Sore throat

☐ Earache

☐ Burning while urinating

☐ Wound/ soft tissue inflammation

☐ Others, namely:

☐ No problems

☐ Do not know

The following concerns your knowledge and opinion about the topic antibiotics:

How do you evaluate the following statements?

1. Antibiotics are effective against bacteria.

strongly agree	agree	rather disagree	disagree	do not know
☐	☐	☐	☐	☐

2. Antibiotics are effective against viruses.

strongly agree	agree	rather disagree	disagree	do not know
☐	☐	☐	☐	☐

3. Penicillin is an antibiotic.

strongly agree	agree	rather disagree	disagree	do not know
☐	☐	☐	☐	☐

4. Paracetamol is an antibiotic.

strongly agree	agree	rather disagree	disagree	do not know
☐	☐	☐	☐	☐

5. Ibuprofen is an antibiotic.

strongly agree	agree	rather disagree	disagree	do not know
☐	☐	☐	☐	☐

6. If an antibiotic is not taken in the way as recommended by the physician, this will increase the risk that pathogens get resistant against this antibiotic.

strongly agree	agree	rather disagree	disagree	do not know
☐	☐	☐	☐	☐

7. Individuals who take antibiotics regularly have a higher risk that their body is getting immuned against antibiotic

strongly agree	agree	rather disagree	disagree	do not know
☐	☐	☐	☐	☐

8. I have already asked my physician for a prescription of antibiotic due to a cold.

strongly agree	agree	rather disagree	disagree	do not know
☐	☐	☐	☐	☐

9. I have antibiotics at home and take it when required.

strongly agree	agree	rather disagree	disagree	do not know
☐	☐	☐	☐	☐

The names of these antibiotics are: ....

10. Usually, I take antibiotic as recommended by the physician or pharmacist.

strongly agree	agree	rather disagree	disagree	do not know
☐	☐	☐	☐	☐

11. I stop taking antibiotic when I feel better.

strongly agree	agree	rather disagree	disagree	do not know
☐	☐	☐	☐	☐

12. If a family member is ill, I share my antibiotic with him/her.

strongly agree	agree	rather disagree	disagree	do not know
☐	☐	☐	☐	☐

13. I do not take antibiotics in general.

strongly agree	agree	rather disagree	disagree	do not know
☐	☐	☐	☐	☐

14. I am concerned about the development of antibiotic resistance.

strongly agree	agree	rather disagree	disagree	do not know
☐	☐	☐	☐	☐

By answering the following questions, please refer to the last time you took antibiotics.

15. I have asked my physician for a prescription at the time.

strongly agree	agree	rather disagree	disagree	do not know
☐	☐	☐	☐	☐

16. I kept the recommended amount of tablets per day.

strongly agree	agree	rather disagree	disagree	do not know
☐	☐	☐	☐	☐

17. I kept the recommended time intervals for the taking.

strongly agree	agree	rather disagree	disagree	do not know
☐	☐	☐	☐	☐

18. I kept the recommended taking duration (e.g. 7 days).

strongly agree	agree	rather disagree	disagree	do not know
☐	☐	☐	☐	☐

19. For which reasons, if you stop the therapy earlier? (Multiple answers are possible):

☐ I felt already better.

☐ I worried about side effects.

☐ I had side effects.

☐ I forgot it.

☐ I had too much stress.

☐ Others:____

20. I suffered under following side effects at the last antibiotic taking (multiple answers are possible):

☐ None

☐ Allergic reaction

☐ Skin rash

☐ Diarrhoea

☐ Sickness

☐ Others:____

☐ Do not know
