# Supplementary material for: Knowledge on Antibiotic Use, Self-Reported Adherence to Antibiotic Intake, and Knowledge on Multi-Drug Resistant Pathogens – Results of a Population-Based Survey in Lower Saxony, Germany
Source: Front Microbiol. 2019 Apr 12;10:776. doi: 10.3389/fmicb.2019.00776 (PMC6473076; doi:10.3389/fmicb.2019.00776)
Supplement: Supplementary file 2 [file Data_Sheet_2.PDF]

Fragebogen zu Antibiotika

In diesem Teil des Fragebogens erkundigen wir uns bei Ihnen nach der Einnahme von Antibiotika. Antibiotika sind Medikamente, die Infektionserreger in ihrem Wachstum bremsen oder abtöten. Infektionen werden durch Krankheitserreger hervorgerufen und gehen häufig mit Fieber einher.

In Deutschland sind Antibiotika rezeptpflichtig und müssen durch einen Arzt/Ärztin verschrieben werden.

1. Bitte erinnern Sie sich an die letzten 12 Monate. Wie häufig wurde Ihnen in diesem Zeitraum ein Antibiotikum verschrieben?

☐ gar nicht ☐ zwei- bis dreimal ☐ weiß nicht

☐ einmal ☐ mehr als viermal

2. Auf Grund welcher Beschwerden haben Sie das letzte Mal Antibiotika verschrieben bekommen? *Mehrfachantworten möglich*

☐ Husten

☐ Halsschmerzen

☐ Ohrenschmerzen

☐ Brennen beim Wasserlassen

☐ Wunde/Weichteilentzündung

☐ weiß nicht

☐ Sonstiges: ____

Wie bewerten Sie die folgenden Aussagen?

3. Antibiotika wirken gegen Bakterien.

☐ Trifft zu ☐ Trifft eher zu

☐ Trifft eher nicht zu ☐ Trifft nicht zu

☐ weiß nicht

4. Antibiotika wirken gegen Viren.

☐ Trifft zu ☐ Trifft eher zu

☐ Trifft eher nicht zu ☐ Trifft nicht zu

☐ weiß nicht

5. Penicillin ist ein Antibiotikum.

☐ Trifft zu ☐ Trifft eher zu

☐ Trifft eher nicht zu ☐ Trifft nicht zu

☐ weiß nicht

6. Paracetamol ist ein Antibiotikum.

- ☐ Trifft zu
- ☐ Trifft eher zu
- ☐ Trifft eher nicht zu
- ☐ Trifft nicht zu
- ☐ weiß nicht

7. Ibuprofen ist ein Antibiotikum.

- ☐ Trifft zu
- ☐ Trifft eher zu
- ☐ Trifft eher nicht zu
- ☐ Trifft nicht zu
- ☐ weiß nicht

8. Wenn ein Antibiotikum nicht so eingenommen wird, wie vom Arzt empfohlen, steigt das Risiko, dass Krankheitserreger gegen dieses Antibiotikum resistent werden. Das heißt, sie werden von dem Antibiotikum nicht mehr abgetötet.

- ☐ Trifft zu
- ☐ Trifft eher zu
- ☐ Trifft eher nicht zu
- ☐ Trifft nicht zu
- ☐ weiß nicht

9. Wer häufiger Antibiotika einnimmt, hat ein höheres Risiko, dass der Körper immun gegen Antibiotika wird. Das heißt, die Antibiotika wirken nicht mehr.

- ☐ Trifft zu
- ☐ Trifft eher zu
- ☐ Trifft eher nicht zu
- ☐ Trifft nicht zu
- ☐ weiß nicht

10. Ich habe meinen Hausarzt auf Grund einer Erkältung schon mal um die Verschreibung eines Antibiotikums gebeten.

- ☐ Trifft zu
- ☐ Trifft eher zu
- ☐ Trifft eher nicht zu
- ☐ Trifft nicht zu
- ☐ weiß nicht

11. Ich habe zu Hause Antibiotika und nehme diese bei Bedarf ein.

- ☐ Trifft zu
- ☐ Trifft eher zu
- ☐ Trifft eher nicht zu
- ☐ Trifft nicht zu
- ☐ weiß nicht

12. Es handelt sich dabei um folgendes Antibiotikum/folgende Antibiotika:

____

Wie bewerten Sie die folgenden Aussagen?

13. Ich nehme Antibiotika in der Regel genau so ein, wie es mir durch Arzt oder Apotheker empfohlen wurde.

☐ Trifft zu ☐ Trifft eher zu
☐ Trifft eher nicht zu ☐ Trifft nicht zu
☐ weiß nicht

14. Ich beende die Einnahme von Antibiotika, sobald es mir besser geht.

☐ Trifft zu ☐ Trifft eher zu
☐ Trifft eher nicht zu ☐ Trifft nicht zu
☐ weiß nicht

15. Wenn ein Angehöriger krank ist, teile ich mein Antibiotikum mit ihm/ihr.

☐ Trifft zu ☐ Trifft eher zu
☐ Trifft eher nicht zu ☐ Trifft nicht zu
☐ weiß nicht

16. Ich nehme grundsätzlich keine Antibiotika ein.

☐ Trifft zu ☐ Trifft eher zu
☐ Trifft eher nicht zu ☐ Trifft nicht zu
☐ weiß nicht

17. Die Entwicklung von Antibiotikaresistenz macht mir Sorgen. (Durch die Resistenz verlieren die Antibiotika ihre Wirksamkeit.)

☐ Trifft zu ☐ Trifft eher zu
☐ Trifft eher nicht zu ☐ Trifft nicht zu
☐ weiß nicht

18. Bei der letzten Antibiotikaeinnahme habe ich unter folgenden Nebenwirkungen gelitten:
Mehrfachantworten möglich

☐ keine
☐ Allergische Reaktion
☐ Hautausschlag
☐ Durchfall
☐ Übelkeit
☐ Sonstiges: ____________________

19. Falls Sie die Therapie frühzeitig beendet habe, aus welchen Gründen? *Mehrfachantworten möglich*

- ☐ weil ich mich bereits besser gefühlt habe
- ☐ weil ich Nebenwirkungen befürchtet habe
- ☐ weil ich Nebenwirkungen hatte
- ☐ weil ich es vergessen habe
- ☐ weil ich zu viel Stress hatte
- ☐ Sonstiges: ____

Wie bewerten Sie die folgenden Aussagen?

Bitte antworten Sie spontan. Beziehen Sie sich bei der Beantwortung der folgenden Fragen auf das letzte Mal, als Sie Antibiotika eingenommen haben.

20. Als mir das letzte Mal ein Antibiotikum verschrieben wurde, habe ich meinen Arzt um die Verschreibung des Antibiotikums gebeten.

☐ Trifft zu ☐ Trifft eher zu
☐ Trifft eher nicht zu ☐ Trifft nicht zu
☐ weiß nicht

21. Ich habe mich an die empfohlene Anzahl der Tabletten pro Tag gehalten.

☐ Trifft zu ☐ Trifft eher zu
☐ Trifft eher nicht zu ☐ Trifft nicht zu
☐ weiß nicht

22. Ich habe mich an die empfohlenen Zeitabstände zur Einnahme gehalten.

☐ Trifft zu ☐ Trifft eher zu
☐ Trifft eher nicht zu ☐ Trifft nicht zu
☐ weiß nicht

23. Ich habe mich an die empfohlene Einnahmedauer (z.B. 7 Tage) gehalten.

☐ Trifft zu ☐ Trifft eher zu
☐ Trifft eher nicht zu ☐ Trifft nicht zu
☐ weiß nicht

24. Gibt es noch etwas, das Sie uns im Rahmen dieses Fragebogens mitteilen möchten?

Fragebogen zu multiresistenten Keimen

Wir möchten Sie zu multiresistenten Keimen, auch bekannt als „Krankenhauskeime“, befragen. Damit meinen wir Krankheitserreger, die gegenüber verschiedenen Antibiotika widerstandsfähig (d.h. resistent) sind. Wenn diese Keime Krankheiten verursachen, müssen die Betroffenen mit besonderen Antibiotika behandelt werden.

1. Haben Sie schon einmal von multiresistenten Keimen gehört?

☐ Ja → weiter zu Frage 2[1]

☐ Nein → Fragebogen beendet

2. Wo oder von wem haben Sie etwas über multiresistente Keime gehört?

	Ja	Nein
Fernsehen	☐	☐
Radio	☐	☐
Zeitung	☐	☐
Familie/Freunde	☐	☐
Internet	☐	☐
Broschüren/Infohefte	☐	☐
Arzt/Ärztin	☐	☐
Krankenhauspersonal	☐	☐
Arbeitskollegen/innen	☐	☐

andere, und zwar ___________________________________

3. Finden Sie das Thema multiresistente Keime wichtig?

☐ sehr wichtig ☐ ziemlich wichtig ☐ mittelmäßig wichtig

☐ wenig wichtig ☐ nicht wichtig

4. Wurde bei Ihnen selbst schon einmal ein multiresistenter Keim festgestellt?

☐ Ja ☐ Nein ☐ weiß nicht

5. Kennen Sie jemanden, bei dem schon einmal ein multiresistenter Keim festgestellt wurde?

☐ Ja → weiter zu Frage 6

☐ Nein → weiter zu Frage 7

[1] Programmierhinweis; für den Teilnehmer nicht sichtbar

6. Wen kennen Sie, bei dem schon einmal ein multiresistenter Keim festgestellt wurde? Mehrere Antworten möglich

- ☐ ein Familienmitglied
- ☐ ein(e) Freund(in)
- ☐ ein(e) Bekannte(r)
- ☐ jemand anderes, und zwar: ______________________________

7. Haben Sie persönlich Angst davor, sich mit multiresistenten Keimen anzustecken?

☐ sehr ☐ ziemlich ☐ mittelmäßig
☐ wenig ☐ nicht

8. Haben Sie Angst davor, dass sich ein Familienangehöriger mit einem multiresistenten Keim ansteckt?

☐ sehr ☐ ziemlich ☐ mittelmäßig
☐ wenig ☐ nicht

9. Machen Ihnen multiresistente Keime als gesamtgesellschaftliches Problem Angst?

☐ sehr ☐ ziemlich ☐ mittelmäßig
☐ wenig ☐ nicht

Im Folgenden möchten wir Ihre Meinung zu multiresistenten Keimen erfassen.

10. Mit multiresistenten Keimen kann man sich nur im Krankenhaus anstecken.

☐ stimme voll zu ☐ stimme zu
☐ stimme eher nicht zu ☐ stimme nicht zu
☐ weiß nicht

11. Multiresistente Keime können nicht behandelt werden.

☐ stimme voll zu ☐ stimme zu
☐ stimme eher nicht zu ☐ stimme nicht zu
☐ weiß nicht

12. Wenn ich die Einnahme eines Antibiotikums sofort bei Besserung der Krankheit beende, trage ich zur Vermeidung von multiresistenten Keimen bei.

☐ stimme voll zu ☐ stimme zu
☐ stimme eher nicht zu ☐ stimme nicht zu
☐ weiß nicht

13. Solange ich einen multiresistenten Keim nur auf der Haut oder Schleimhaut trage, ist das für mich erst einmal nicht gefährlich.

- ☐ stimme voll zu
- ☐ stimme zu
- ☐ stimme eher nicht zu
- ☐ stimme nicht zu
- ☐ weiß nicht

Im Folgenden möchten wir wissen, was Ihrer Meinung nach zu der Verbreitung von multiresistenten Keimen beiträgt.

14. Welche Ursachen sind für die Verbreitung von multiresistenten Keimen Ihrer Meinung nach wichtig?

	sehr wichtig	ziemlich wichtig	mittelmäßig wichtig	wenig wichtig	nicht wichtig	weiß nicht
unsachgemäße Antibiotikaeinnahmen in der Bevölkerung	☐	☐	☐	☐	☐	☐
unsachgemäßer Antibiotikaeinsatz in der Tierzucht	☐	☐	☐	☐	☐	☐
mangelnde Hygiene im medizinischen Bereich generell	☐	☐	☐	☐	☐	☐
mangelnde Händehygiene des Krankenhaus-Personals/der Ärzte	☐	☐	☐	☐	☐	☐
mangelnde Händehygiene in der Bevölkerung	☐	☐	☐	☐	☐	☐
mangelnde Bettenkapazität in den Krankenhäusern	☐	☐	☐	☐	☐	☐
zu wenig wirksame Medikamente	☐	☐	☐	☐	☐	☐

Gibt es Ihrer Meinung nach für die Verbreitung von multiresistenten Keimen andere Ursachen? Wenn ja, welche?

Wie wichtig ist die von Ihnen genannte Ursache?

	sehr wichtig	ziemlich wichtig	mittelmäßig wichtig	wenig wichtig	nicht wichtig	weiß nicht
Texteintrag	☐	☐	☐	☐	☐	☐

In den folgenden Fragen geht es um das Thema Tierzucht.

15. Haben Sie oder eine Person, die in Ihrem Haushalt lebt, beruflich Umgang mit Nutztieren, z.B. als Landwirt/in, Tierarzt/-ärztin oder in der Fleischverarbeitung?

- ☐ Ja, ich selbst
- ☐ Ja, eine andere Person, die in meinem Haushalt lebt
- ☐ Nein

16. Politiker sind dafür verantwortlich, dass der Einsatz von Antibiotika in der Tierzucht reduziert wird

- ☐ stimme voll zu
- ☐ stimme zu
- ☐ stimme eher nicht zu
- ☐ stimme nicht zu
- ☐ weiß nicht

17. Landwirte sind dafür verantwortlich, dass der Einsatz von Antibiotika in der Tierzucht reduziert wird

- ☐ stimme voll zu
- ☐ stimme zu
- ☐ stimme eher nicht zu
- ☐ stimme nicht zu
- ☐ weiß nicht

18. Verbraucher sind dafür verantwortlich, dass der Einsatz von Antibiotika in der Tierzucht reduziert wird

- ☐ stimme voll zu
- ☐ stimme zu
- ☐ stimme eher nicht zu
- ☐ stimme nicht zu
- ☐ weiß nicht

19. Ich bin bereit, mehr Geld für Fleisch auszugeben (vergleichbar mit den Kosten für Bioprodukte), wenn dies zu einem reduzierten Einsatz von Antibiotika führt.

- ☐ stimme voll zu
- ☐ stimme zu
- ☐ stimme eher nicht zu
- ☐ stimme nicht zu
- ☐ weiß nicht

In den folgenden drei Fragen geht es um das Thema Verbreitung von Antibiotikaresistenzen und Verantwortung im Gesundheitssystem.

20. Jeder einzelne ist dafür verantwortlich, durch eine korrekte Einnahme von Antibiotika die Verbreitung von multiresistenten Keimen zu verringern.

- ☐ stimme voll zu
- ☐ stimme zu
- ☐ stimme eher nicht zu
- ☐ stimme nicht zu
- ☐ weiß nicht

21. Ärzte und Pflegepersonal sind dafür verantwortlich, eine Weiterverbreitung von multiresistenten Keimen im Gesundheitssystem zu bekämpfen.

- ☐ stimme voll zu
- ☐ stimme zu
- ☐ stimme eher nicht zu
- ☐ stimme nicht zu
- ☐ weiß nicht

22. Politiker sind dafür verantwortlich, eine Weiterverbreitung von multiresistenten Keimen im Gesundheitssystem zu bekämpfen.

- ☐ stimme voll zu
- ☐ stimme zu
- ☐ stimme eher nicht zu
- ☐ stimme nicht zu
- ☐ weiß nicht

Zum Abschluss des Fragebogens folgen zwei Fallbeispiele.

<u>Fallbeispiel 1</u>

23. Ihr Nachbar, ein alleinlebender Senior, benötigt etwas Hilfe und sie kaufen schon seit ein paar Monaten regelmäßig für ihn ein. Nach einem Krankenhausaufenthalt berichtet er Ihnen, er habe sich mit einem Krankenhauskeim angesteckt. Wie würden Sie sich verhalten?

	stimme voll zu	stimme zu	stimme eher nicht zu	stimme nicht zu
Ich verhalte mich genau wie vorher.	☐	☐	☐	☐
Ich wasche mir nach dem Besuch gründlich die Hände.	☐	☐	☐	☐
Ich desinfiziere mir nach dem Besuch die Hände.	☐	☐	☐	☐
Ich wechsle nach dem Besuch meine Kleidung.	☐	☐	☐	☐
Meine Kinder dürfen den Nachbarn jetzt nicht mehr besuchen.	☐	☐	☐	☐
Ich stelle ihm die Einkäufe jetzt nur in den Flur und vermeide näheren Kontakt.	☐	☐	☐	☐
Ich vermeide den Nachbarn jetzt komplett.	☐	☐	☐	☐
Ich habe Angst davor, mich anzustecken.	☐	☐	☐	☐

Fallbeispiel 2

24. Ihre Arbeitskollegin, mit der Sie das Büro teilen und zum Teil die gleichen Gegenstände nutzen, teilt Ihnen nach einem Krankenhausaufenthalt mit, dass sie sich mit einem Krankenhauskeim infiziert hat. Wie würden Sie sich verhalten?

	stimme voll zu	stimme zu	stimme eher nicht zu	stimme nicht zu
Ich verhalte mich genau wie vorher.	☐	☐	☐	☐
Ich wasche mir häufig die Hände.	☐	☐	☐	☐
Ich desinfiziere mir häufig die Hände.	☐	☐	☐	☐
Ich wechsle nach der Arbeit meine Kleidung.	☐	☐	☐	☐
Ich vermeide Körperkontakt zu ihr.	☐	☐	☐	☐
Ich vermeide den Kontakt mit gemeinsam berührten Materialien.	☐	☐	☐	☐
Ich bitte meinen Vorgesetzten um Versetzung in ein anderes Büro/einen anderen Arbeitsbereich.	☐	☐	☐	☐
Ich habe Angst davor, mich anzustecken.	☐	☐	☐	☐
