# Supplementary material for: Knowledge on Antibiotic Use, Self-Reported Adherence to Antibiotic Intake, and Knowledge on Multi-Drug Resistant Pathogens – Results of a Population-Based Survey in Lower Saxony, Germany
Source: Front Microbiol. 2019 Apr 12;10:776. doi: 10.3389/fmicb.2019.00776 (PMC6473076; doi:10.3389/fmicb.2019.00776)
Supplement: Supplementary file 3 [file Data_Sheet_3.PDF]

# Questionnaire on MDR pathogens in HaBIDS

We would like to ask you about multidrug-resistant pathogens, also known as “hospital germs”. Thereby we think about pathogens, which are resistant towards different antibiotics. Concerned persons have to treat with special antibiotics, if this pathogens cause diseases.

1. Have you ever heard of multidrug-resistant pathogens?

☐ Yes [1]     ☐ No [2]

MRE2 Where or from who have you heard something about multidrug-resistant pathogens?

| | Yes 1 | No 0 |
|---|---|---|
| 1Television | ☐ | ☐ |
| 2 Radio | ☐ | ☐ |
| 3 Newspaper | ☐ | ☐ |
| 4 Family/friends | ☐ | ☐ |
| 5 Internet | ☐ | ☐ |
| 6 Information brochure | ☐ | ☐ |
| 7 Doctor | ☐ | ☐ |
| 8 Hospital staff | ☐ | ☐ |
| 9 Colleague | ☐ | ☐ |
| 10 others, namely | | |

MRE2_andere________________________________

MRE3 Do you consider the topic multidrug-resistant pathogens as important?

☐ 4 very important     ☐ 3 quite important     ☐ 2 moderately important

☐ 1 less important     ☐ 0 not important

MRE4 Have you been infected or a carrier of a multidrug-resistant pathogen at least once before?

☐ 1Yes     ☐ 0No     ☐ 90 do not know

5. Do you know someone who had a multidrug-resistant pathogen before?

☐ 1Yes     ☐ 0No

→ weiter zu Frage 0     → weiter zu Frage 0

[1] Programmierhinweis; für den Teilnehmer nicht sichtbar

MRE6 Who? Several answers possible

- ☐ 1 a family member
- ☐ 2 a friend
- ☐ 3 An acquaintance
- ☐ other Other, namely: ________________________________________

The following questions concern your worries about multidrug-resistant pathogens

MRE7Are you personally afraid of infection with multidrug-resistant pathogens?

☐ 4very ☐ 3quite ☐ 2moderately
☐ 1less ☐ 0not

MRE8Are you afraid that a family member infect with a multidrug-resistant pathogen?

☐ 4very ☐ 3quite ☐ 2moderately
☐ 1less ☐ 0not

MRE9Are you frightened that multidrug-resistant pathogens is a social problem?

☐ 4very ☐ 3quite ☐ 2moderately
☐ 1less ☐ 0not

Below we would like to record your opinion about resistant pathogens.

MRE10You can only acquire multidrug-resistant pathogens in hospital.

☐ 4totally agree ☐ 3agree
☐ 2rather disagree ☐ 1do not agree
☐ 90do not know

MRE11 Multidrug-resistant pathogens cannot be treated.

☐ 4totally agree ☐ 3agree
☐ 2rather disagree ☐ 1do not agree
☐ 90do not know

MRE12If I stop taking antibiotics immediately as soon as I feel better, I contribute to avoiding multi-drug resistant pathogens.

☐ 4totally agree ☐ 3agree

☐ 2rather disagree ☐ 1do not agree

☐ 90do not know

MRE13As long as multi-drug resistant pathogens are only on my skin or mucous membrane, it is not dangerous for me.

☐ 4totally agree ☐ 3agree

☐ 2rather disagree ☐ 1do not agree

☐ 90do not know

Below we want to know, what contributes to the distribution of resistant pathogens in your opinion.

MRE14Which causes are important for distribution of multidrug-resistant pathogens?

| | very important4 | quite important3 | moderately important2 | less important1 | not important0 | Do not know90 |
|---|---|---|---|---|---|---|
| 1improper antibiotic intake in population | ☐ | ☐ | ☐ | ☐ | ☐ | ☐ |
| 2improper use of antibiotic in livestock breeding | ☐ | ☐ | ☐ | ☐ | ☐ | ☐ |
| 3lacking hygiene in medical sector in general | ☐ | ☐ | ☐ | ☐ | ☐ | ☐ |
| 4lacking hand hygiene from medical health workers | ☐ | ☐ | ☐ | ☐ | ☐ | ☐ |
| 5lacking hand hygiene in society | ☐ | ☐ | ☐ | ☐ | ☐ | ☐ |
| 6lack of bed capacity in hospitals | ☐ | ☐ | ☐ | ☐ | ☐ | ☐ |
| 7too less effective medicaments | ☐ | ☐ | ☐ | ☐ | ☐ | ☐ |

MRE14_andere Are there other causes which are responsible for distribution of multidrug-resistant pathogens in your opinion? If so, what are they?

MRE14_andere2 How important is this cause?

| | very important 4 | quite important 3 | moderately important 2 | less important 1 | not important 0 | do not know 90 |
|---|---|---|---|---|---|---|
| 9 Texteintrag von MRE14_andere | ☐ | ☐ | ☐ | ☐ | ☐ | ☐ |

The following questions concern the livestock breeding.

MRE15 Do you or another person in your household have occupational contact with live stocks, e.g. as farmer, veterinary or in meat processing?

☐ 1 Yes, myself

☐ 2 Yes, another person who lives in my household

☐ 0 No

MRE16 Politicians are responsible for reducing the use of antibiotics in livestock breeding.

☐ 4 totally agree

☐ 3 agree

☐ 2 rather disagree

☐ 1 do not agree

☐ 90 do not know

MRE17 Farmers are responsible for reducing the use of antibiotics in livestock breeding.

☐ 4 totally agree

☐ 3 agree

☐ 2 rather disagree

☐ 1 do not agree

☐ 90 do not know

MRE18 Consumers are responsible for reducing the use of antibiotics in livestock breeding.

☐ 4 totally agree

☐ 3 agree

☐ 2 rather disagree

☐ 1 do not agree

☐ 90 do not know

MRE19I am willing to spend more money for meat (comparable with costs for organic products), if this leads to reducing the use of antibiotics.

- ☐ 4totally agree
- ☐ 3agree
- ☐ 2rather disagree
- ☐ 1do not agree
- ☐ 90do not know
- ☐ 5I subsist vegetarian and vegan.

The following three questions concern who is responsible to limit the spread of antibiotic resistant pathogens in the health care system.

MRE20Each individual has a responsibility, to correctly take antibiotics, to reduce the spread of multidrug-resistant pathogens.

- ☐ 4totally agree
- ☐ 3agree
- ☐ 2rather disagree
- ☐ 1do not agree
- ☐ 90do not know

MRE21Doctors and care staff are responsible for reducing/combating spread of multidrug-resistant pathogens in the health care system.

- ☐ 4totally agree
- ☐ 1agree
- ☐ 2rather disagree
- ☐ 3do not agree
- ☐ 90do not know

MRE22Politicians are responsible for reducing/combating spread of multidrug-resistant pathogens in the health care system.

- ☐ 4totally agree
- ☐ 3agree
- ☐ 2rather disagree
- ☐ 1do not agree
- ☐ 90do not know

The following two case examples conclude the questionnaire:

Case 1

MRE23 Your neighbour, an elderly living alone, needs some help and you have been shopping for him for a couple of months. After a hospital stay, he tells you that he has become infected with a hospital acquired pathogen. How would you behave with him?

| | Fully agree4 | Agree3 | Rather disagree2 | Disagree1 |
|---|---|---|---|---|
| 1 I behave just like before | ☐ | ☐ | ☐ | ☐ |
| 2 I wash my hands thoroughly after visiting him | ☐ | ☐ | ☐ | ☐ |
| 3 I disinfect my hands after visiting him | ☐ | ☐ | ☐ | ☐ |
| 4 I change my clothes after visiting him | ☐ | ☐ | ☐ | ☐ |
| 5 My children are not allowed to visit him anymore | ☐ | ☐ | ☐ | ☐ |
| 6 I now put the daily purchases in the hallway and avoid close contact | ☐ | ☐ | ☐ | ☐ |
| 7 I avoid the neighbor completely now | ☐ | ☐ | ☐ | ☐ |
| 8 I am afraid of getting infected | ☐ | ☐ | ☐ | ☐ |

Case 2

MRE24 Your co-worker, with whom you share the office and use some of the same items, tells you after a stay in the hospital that she has become infected with a hospital acquired pathogen. How would you behave with him?

| | Fully agree4 | Agree3 | Rather disagree2 | Disagree1 |
|---|---|---|---|---|
| 1 I behave like before | ☐ | ☐ | ☐ | ☐ |
| 2 I wash my hands frequently | ☐ | ☐ | ☐ | ☐ |
| 3 I disinfect my hands frequently | ☐ | ☐ | ☐ | ☐ |
| 4 I change my clothes after work | ☐ | ☐ | ☐ | ☐ |
| 5 I avoid body contact with her | ☐ | ☐ | ☐ | ☐ |
| 6 I avoid contact with the materials we share | ☐ | ☐ | ☐ | ☐ |
| 7 I ask my supervisor for a transfer to another office / workspace | ☐ | ☐ | ☐ | ☐ |
| 8 I am afraid of getting infected | ☐ | ☐ | ☐ | ☐ |
